# Supplementary figures and images for: Size and structure of the sequence space of repeat proteins
Source: PLoS Comput Biol. 2019 Aug 15;15(8):e1007282. doi: 10.1371/journal.pcbi.1007282 (PMC6733475; doi:10.1371/journal.pcbi.1007282)

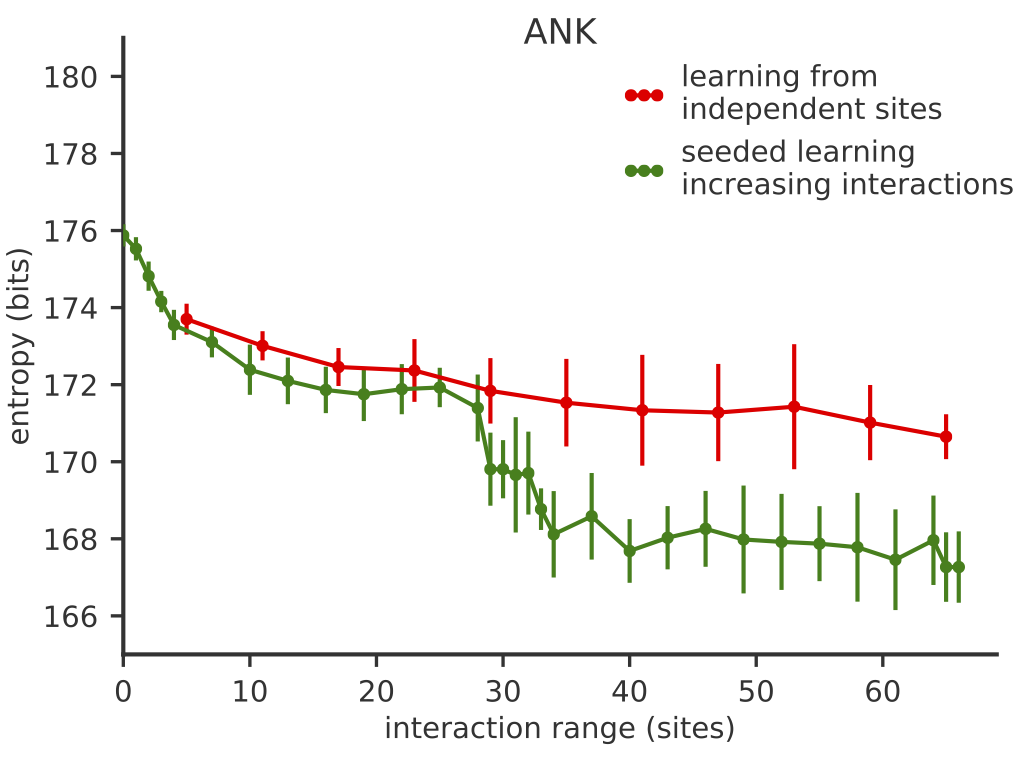

Supplement: S1 Fig — Entropy as a function of the maximum linear interaction range W along the sequence. Green curve: entropy of the ANK family with error bars calculated as standard deviations over 10 model learning realizations, where models are learned by incrementally adding more interaction terms as W is increased, taking the model learned at W − 1 as initial condition. This plot is the same as in Fig 3A but with the different error bar estimates, showing that our results are robust to the details of error estimation. Red curve: entropy obtained after de novo learning for each W, starting from a non-interacting model as initial condition. With those initial conditions the learning gets stuck, leading to systematically overestimating the entropy and missing the second entropy drop at W = L − 1. See Section for details of the learning and entropy estimation procedure. (TIFF) [file pcbi.1007282.s001.tiff]

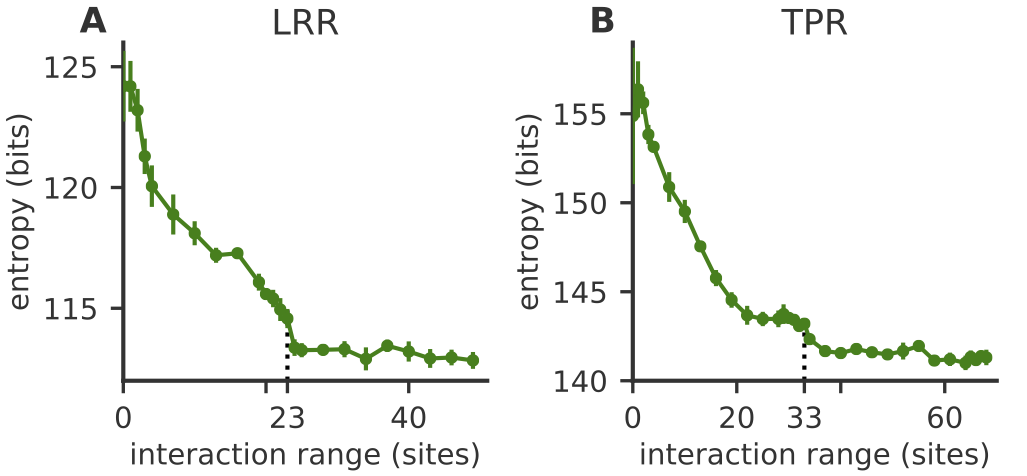

Supplement: S2 Fig — Entropy of the LRR (A) and TPR (B) family as a function of the maximum interaction distance W along the sequence. The entropy of the model decreases as a more interactions are added and they constrain the space of possible sequences. As with ANK, the entropy first drops, plateaus, then drops again at the distance corresponding to homologous positions along the two repeats (W = L − 1 = 23 for LRR, and 33 for TPR, dashed line). This second drop indicates that there is a typical distance along the sequence, corresponding to the repeat length, where interactions due to structural properties constrain the sequence ensemble. The error bars are estimated approximately from errors in learning (see Section). Entropies are averaged over 5 realizations of the learning and entropy estimation procedure. (TIFF) [file pcbi.1007282.s002.tiff]

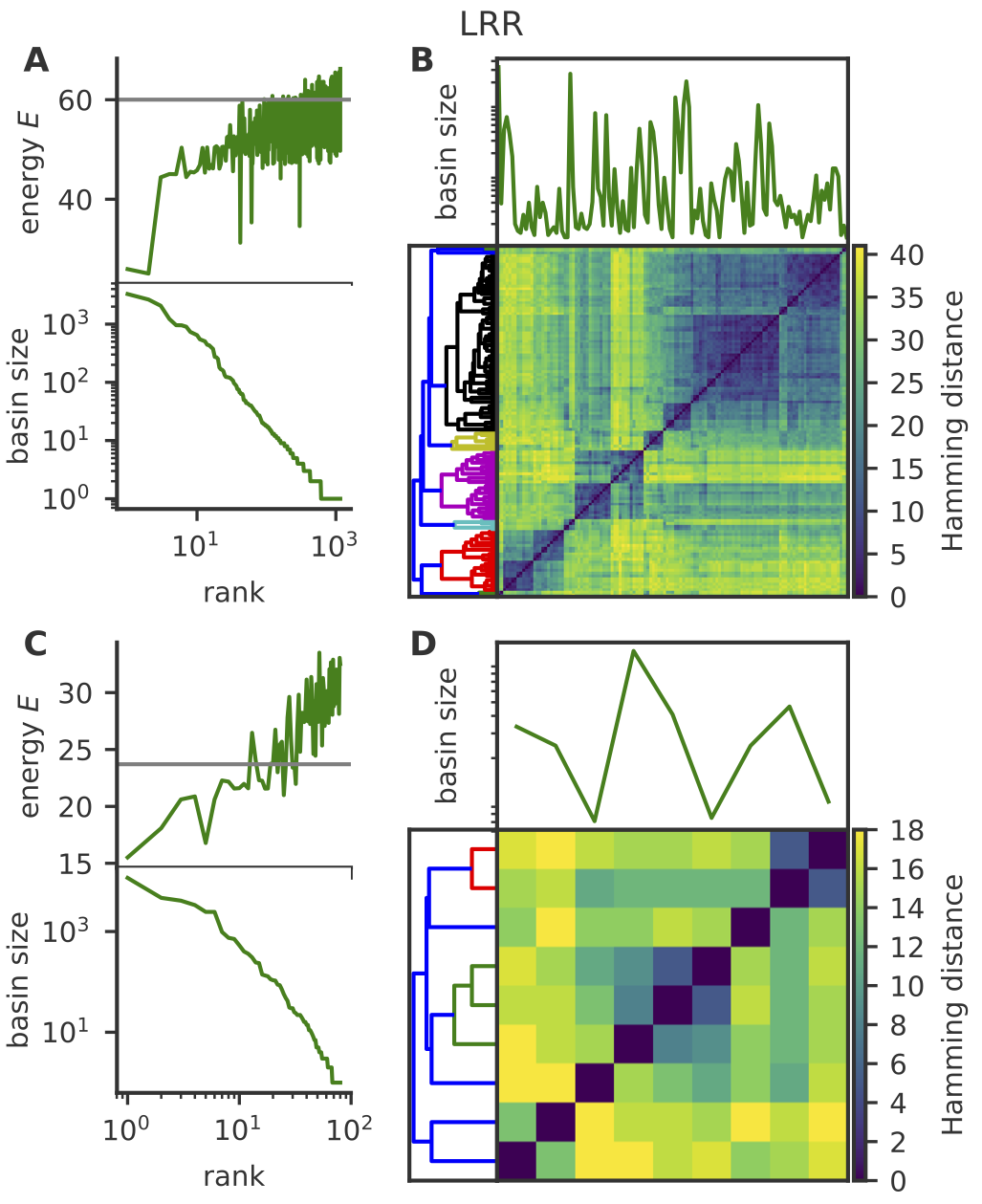

Supplement: S3 Fig — Energy minima were obtained by zero-temperature dynamics. Sequences falling into a given minimum with these dynamics define its basin of attraction. A, bottom) rank-frequency plot of the sizes of the basins of attraction. A, top) energy minimum of each basin. Gray line shows the energy of the consensus sequence B) Pairwise Hamming distances between energy minima, organised by hierarchical clustering. The panel right above the matrix shows the the size of the basins relative to the minima corresponding to the entries of the distance matrix. C and D) Same analysis as A) and B), but for single LRR repeats. (TIFF) [file pcbi.1007282.s003.tiff]

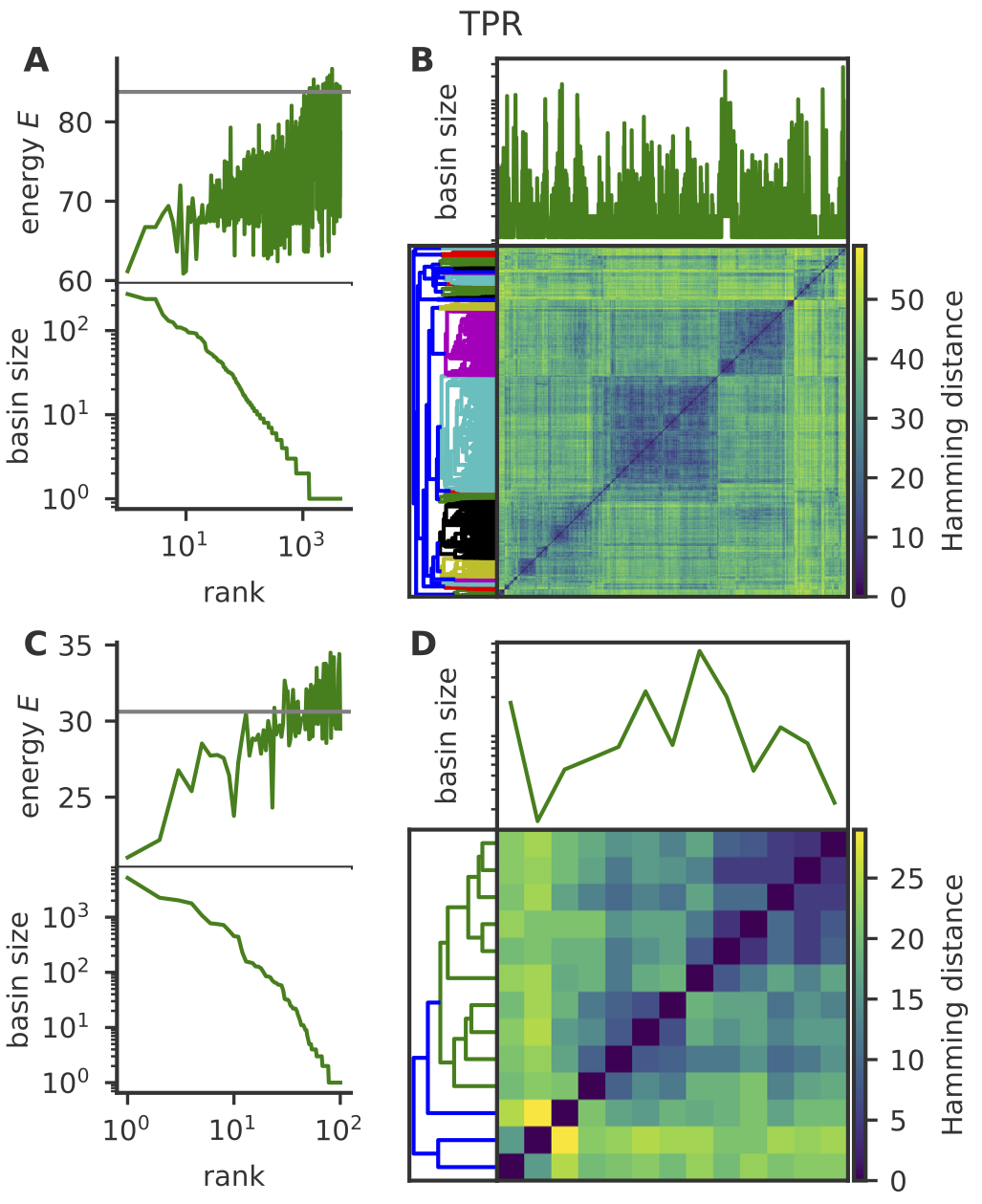

Supplement: S4 Fig — Energy minima were obtained by zero-temperature dynamics. Sequences falling into a given minimum with these dynamics define its basin of attraction. A, bottom) rank-frequency plot of the sizes of the basins of attraction. A, top) energy minimum of each basin. Gray line shows the energy of the consensus sequence B) Pairwise Hamming distances between energy minima, organised by hierarchical clustering. The panel right above the matrix shows the the size of the basins relative to the minima corresponding to the entries of the distance matrix. C and D) Same analysis as A) and B), but for single TPR repeats. (TIFF) [file pcbi.1007282.s004.tiff]

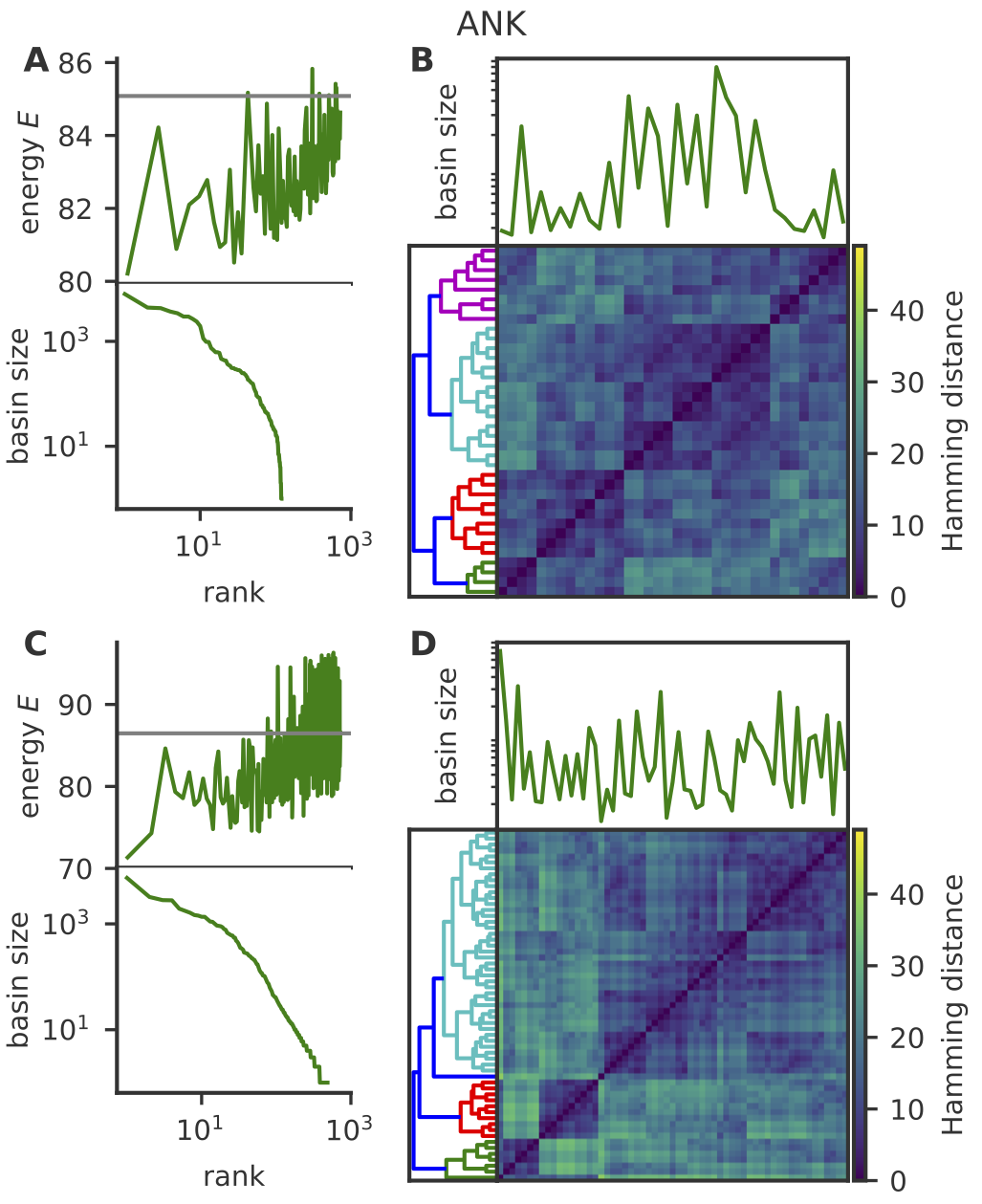

Supplement: S5 Fig — Local minima were obtained by performing a zero-temperature Monte-Carlo simulation with the energy function in Eq (2) with non-zero Jij within linear interaction range W, starting from initial conditions corresponding to naturally occurring sequences of pairs of consecutive ANK repeats, for W = 3 (A and B) and W = 10 (C and D). See Fig 5 for the full model (W = 2L). A and C, bottom: Rank-frequency plot of basin sizes, where basins are defined by the set of sequences falling into a particular minimum. A and C, top: energy of local minima vs the size-rank of their basin. Gray line indicates the energy of the consensus sequence, for comparison. B and D: Pairwise distance between the minima with the largest basins (comprising 90% of natural sequences), organised by hierarchical clustering. The panel right above the matrix shows the size of the basins relative to the minima corresponding to the entries of the distance matrix. The block structure starts emerging as interactions are turned on (D versus B). (TIFF) [file pcbi.1007282.s005.tiff]

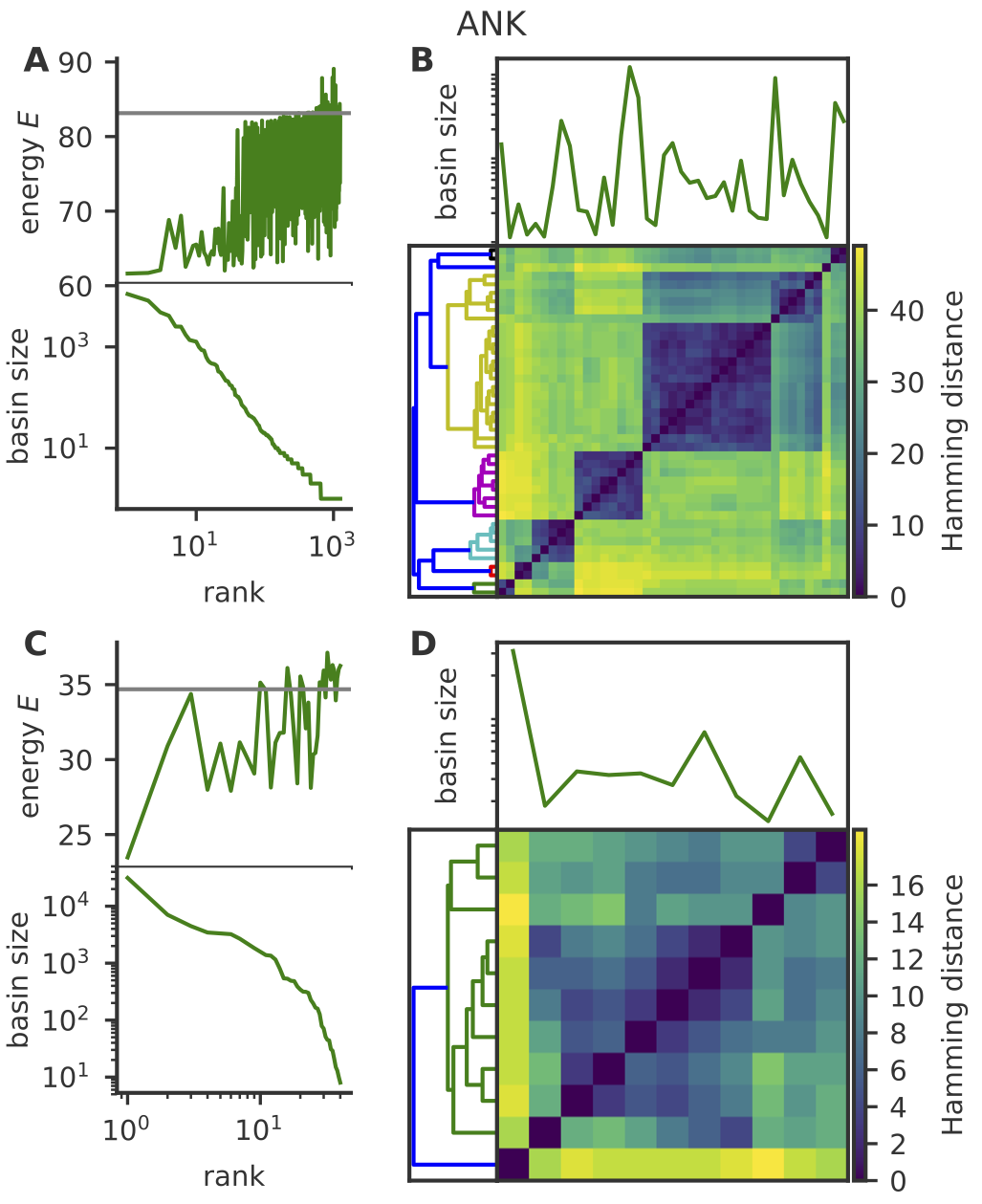

Supplement: S6 Fig — Energy minima were obtained by zero-temperature dynamics starting from sequences generated in silico from Efull. Sequences falling into a given minimum with these dynamics define its basin of attraction. A, bottom) rank-frequency plot of the sizes of the basins of attraction. A, top) energy minimum of each basin. Gray line shows the energy of the consensus sequence B) Pairwise Hamming distances between energy minima, organised by hierarchical clustering. The panel right above the matrix shows the size of the basins relative to the minima corresponding to the entries of the distance matrix. C and D) Same analysis as A) and B), but for single ANK repeats. (TIFF) [file pcbi.1007282.s006.tiff]
